# Rabies-Specific Antibodies: Measuring Surrogates of Protection against a Fatal Disease

**DOI:** 10.1371/journal.pntd.0000595

**Published:** 2010-03-09

**Authors:** Susan M. Moore, Cathleen A. Hanlon

**Affiliations:** Rabies Laboratory, Kansas State Veterinary Diagnostic Laboratory, Kansas State University, Manhattan, Kansas, United States of America; George Washington University, United States of America

## Abstract

Antibodies play a central role in prophylaxis against many infectious agents. While neutralization is a primary function of antibodies, the Fc- and complement-dependent activities of these multifunctional proteins may also be critical in their ability to provide protection against most viruses. Protection against viral pathogens in vivo is complex, and while virus neutralization—the ability of antibody to inactivate virus infectivity, often measured in vitro—is important, it is often only a partial contributor in protection. The rapid fluorescent focus inhibition test (RFFIT) remains the “gold standard” assay to measure rabies virus–neutralizing antibodies. In addition to neutralization, the rabies-specific antigen-binding activity of antibodies may be measured through enzyme-linked immunosorbent assays (ELISAs), as well as other available methods. For any disease, in selecting the appropriate assay(s) to use to assess antibody titers, assay validation and how they are interpreted are important considerations—but for a fatal disease like rabies, they are of paramount importance. The innate limitations of a one-dimensional laboratory test for rabies antibody measurement, as well as the validation of the method of choice, must be carefully considered in the selection of an assay method and for the interpretation of results that might be construed as a surrogate of protection.

## Introduction

Whether an animal control worker wants to determine if a rabies vaccine booster is necessary to establish an acceptable pre-exposure status, or a physician is considering the causes of encephalitis in a child, or the owner of an immunologically compromised dog is worried that the dog's response to rabies vaccination will not be sufficient to pass a serological test allowing them to travel to a rabies-free area, or a researcher is trying to determine if the rabies vaccine-bait response is adequate in a raccoon population, or one needs to assign a potency value to a rabies immune globulin product, all demand an accurate assessment based on the measurement of circulating antibodies. In each of these situations the measurement or simply the detection of rabies-specific virus-neutralizing or other antibodies will help resolve the question. However, just as the circumstances in each of these scenarios are different, the specifics of the method chosen to measure antibodies, the regulatory requirements of the testing, and the purpose of testing in each of these situations are different. Antibodies arise from the humoral immune response to rabies antigens, the process of which is controlled by many factors, including the amount of antigen, route of delivery, the expression and involvement of major histocompatibility complex (MHC) genes, and the health status of the individual, among others. An understanding of the host immune response, including the immunoglobulin (Ig) subclass (type) and the kinetics and longevity of the response, is necessary to obtain the measure or degree of rabies immunity.

Initially, measurement of rabies virus neutralizing antibodies (RVNA) was performed in vivo using the mouse neutralization test (MNT). Subsequently, the rapid fluorescent focus inhibition test (RFFIT) was established as the “gold standard” in vitro test [1]. Methods for measuring rabies immunity vary with regard to the humoral component measured (i.e., the Ig subclass or functional activity) and performance characteristics (i.e., specificity or sensitivity), as well as the cost and complexity of the method. Understanding each of these unique factors is essential in the selection and proper use of the method and is equally critical in the interpretation of the results derived from the methods. Moreover, regulations (e.g., from the United States Food and Drug Administration (USFDA), European Union, World Organization for Animal Health) that require validated and approved test methods for measuring the generation of rabies virus antibodies are relevant in pet transport and in rabies biologics production and evaluation, for use in both humans and animals. In this review, we discuss the following (1) the role of rabies-specific antibodies in disease prevention, (2) the methods that can be used for detection and measurement, and (3) the considerations and current requirements for method standardization and validation.

## Methods

A review of the literature was conducted using the online database PubMed from 1975 to 2008 with US National Library of Medicine medical subject headings (MeSH). Reference lists of selected articles and reviews were also individually researched. In addition, unpublished rabies serology data from the Kansas State University Rabies Laboratory were reviewed.

### The Role of Rabies-Specific Antibodies in Disease Prevention

Animal models of protection against rabies have demonstrated the essential role of RVNA [2],[3]. Indeed, RVNA alone can result in viral clearance from the central nervous system (CNS) of experimentally infected mice [4]. Most rabies-specific antibodies are directed to epitopes on the rabies virus glycoprotein, although some may specifically recognize the nucleoprotein [5],[6]. How rabies-specific antibodies neutralize the virus is not entirely understood in terms of what specific epitopes or Ig subclasses are involved [7],[8]. A single rabies virion can bind up to 1,000 molecules of some antiglycoprotein monoclonal antibodies without being neutralized, suggesting that virus neutralization probably involves more than simply antibody binding to virion epitopes [7],[8]. It can be assumed that antibodies that neutralize virus in vitro are more efficient in the process that leads to protection against virus infection in vivo than antibodies that do not neutralize virus in vitro. The Fc portion of antibodies alone has specific biological functions, including activation of antigen-presenting cells and the complement cascade. As such, whole IgG is expected to be more potent than the epitope-specific F(ab)^2^ portions of neutralizing antibodies [9]. With the development of molecular techniques for engineering the specificity of antibody Fab fragments and intact monoclonal antibodies for diagnostic and therapeutic purposes, the importance of understanding how antibodies neutralize rabies virus mechanistically becomes all the more important. Because it is difficult in practical terms to predict the ability of a polyclonal or monoclonal antibody preparation to neutralize virus by measurements of antibody avidity or affinity alone, neutralization activity must be determined first by some means in vitro even if only to provide an estimate of in vivo activity. That said, a preparation of rabies-specific antibodies that demonstrates potent neutralization in vitro may still not protect in vivo [10].

The rabies virus is highly neurotropic. Within the CNS, rabies virus infection develops for the most part undetected by the adaptive immune responses of the host until the infection is in its final stages. In addition to an infection that develops within this immune-privileged site, the rabies virus is able to subvert any host immune response that is mounted and thus the outcome of infection is fatal in nearly 100% of cases. In contrast, a rabies vaccine is able to stimulate high levels of circulating RVNA. This is why immunity following exposure to rabies virus in an unvaccinated individual, initiated with prompt wound cleansing to reduce viral load at the site of exposure and administration of rabies-specific Ig followed by a series of rabies vaccinations, is virtually 100% effective in preventing infection. Passive protection with rabies Ig (RIG) is critical to immediately neutralize a majority of infectious virions, keeping the virus from spreading while waiting for induction of the host's adaptive immune response. In general, rabies vaccines historically are killed whole-virus vaccines, which are expected to promote the development of anti-rabies virus-specific antibodies along with a CD4^+^ T lymphocyte response, which includes cytokine production. Each individual's polyclonal antibody response to rabies vaccination is a unique mix of specificities to rabies virus antigens. Although the majority of individuals develop a variety of measurable antibodies in response to pre- or postexposure vaccination, there may be substantial variation in the neutralizing activity and quantity of RVNA produced.

Antibodies develop out of intrinsic host responses and in response to extrinsic factors such as the amount of antigen given, type of antigen, and route of exposure or vaccination. For example, it has been demonstrated that the more potent the vaccine the higher the levels of RVNA that are induced in human subjects [11]. Other studies have demonstrated that the type of vaccine and route of vaccination affect the level of antibodies at both 14 days and one year after vaccination [12]. Also, a T helper type 2 (Th2) immune response to killed vaccine is inherently different from activation of a T helper type 1 (Th1) immunity by a live virus vaccine. In addition to host factors such as age and general health status, an individual's adaptive immune response requires the binding of foreign peptides to the MHC molecule on antigen-presenting cells and stimulation of a corresponding T cell clone by that particular MHC–peptide combination. The diversity of the genes in the MHC complex, consisting of hundreds of alleles, influences the variation in the immune response to vaccination [13]. Humoral (Th2) and cellular (Th1) type immune responses are largely directed by the production of cytokines, including maturation of the antibody response and generation of Ig subclass. A dichotomy of responses in rabies vaccination among humans to high or good responders and poor or low responders has also been observed [14]. Predictions regarding the longevity of the RVNA response can be made on the basis of the level and timing of the peak response to vaccination [12],[14].

### Methods for the Detection and Measurement of Rabies Virus Antibodies

Methods available for the detection and measurement of rabies virus–specific antibodies are either antigen-binding assays or virus-neutralization assays. In antigen-binding assays, antibodies in serum or cerebrospinal fluid (CSF) are detected, quantified, and characterized by their ability to bind to various rabies virus antigens, from whole virus, purified subunits, or specific peptides that mimic epitopes. Such assays determine the affinity, avidity, and specificity of binding antibodies. Commonly, these methods involve fixing the antigen to a solid surface, i.e., tube, plate, or bead. The interaction between the antibody and the antigen is then visualized and quantitated by various detection systems that involve color development through an enzyme–substrate reaction or binding of a secondary antibody, conjugated to a fluorescent marker or staphylococcal protein A/G, to the primary antibody bound to the antigen. These assays can be used to measure antibodies that react to internal viral proteins, either structural or enzymatic, as well as to estimate levels of antibodies that bind to the external proteins against which neutralizing antibodies are directed. In contrast, modern virus neutralization assays are cell-based assays that detect the functional activity of antibodies in the serum or CSF against live virus. The mechanism of virus neutralization in cell culture (an in vitro assay) depends upon the interaction between the virion epitope and the paratope (the specific counteracting site) of the antibody, and may also be influenced by receptor characteristics of the cells upon which an assay is performed [15]. Evidence indicates that the virus infects cells of neuronal origin using the receptors nicotinic acetylcholine receptor, the low-affinity nerve growth factor receptor, or the neural cell adhesion molecule, while infecting non-CNS cells by use of other unidentified receptors [16],[17]. The outcome of a virus-neutralization assay is based on a measurement of virus growth in cell culture, i.e., defining whether virus escapes neutralization or not. Binding assays, on the other hand, measure a different set of characteristics of the rabies-specific Ig response compared with neutralization assays. Hence, the results from binding assays are not, a priori, directly comparable to neutralization results [7]. With appropriate validation and suitability for the intended purpose, binding assays can be used to determine the presence or absence of antigen-specific antibodies, and in some cases Ig subclasses, that could be used as an approximation or confirmation of the neutralizing antibody response. The purpose of testing will determine which methods are most appropriate. For example, detection of specific rabies virus antibodies in the CSF is diagnostic of rabies infection, whether the test performed is one of the binding assays (such as to fixed whole virus on a slide that is detected with anti-species IgG or IgM) or a neutralization assay (such as the RFFIT). Antibody activity detected in sera may be an indication of prior vaccination or, if present with clinical signs of rabies, of active infection or even, in very rare cases, prior exposure to rabies virus that resulted in no clinical infection [18] or in survival of the infection by the individual [19]. To fully define immunity to rabies requires a highly specific assay and the ability to detect extremely low levels of antibodies, and often of specific subclasses such as IgM and IgG. Binding assays can more easily be devised to achieve these requirements through use of specific, pure antigen and robust detection (readout) systems. Nonetheless, a measure of protection against rabies infection is best approximated by a virus neutralization test. Since experimental virus challenge methods will never be performed in humans, surrogate experimental animal models of protection based on field observations and the amount and duration of serum neutralizing antibodies measured by in vitro methods are used.

By changing various components, such as virus strain, detection system, etc., of the assay, it is possible to custom design a “fit-for-purpose” assay. For example, detection of RVNA in a particular colony of bat species associated with a rabies virus variant may be “specifically” measured using, in the in vitro assay, the suspected rabies virus variant in circulation among the bats as the challenge virus. The sensitivity of a test method may be adjusted by varying the amount of antigen or challenge virus dose to more precisely define and determine low levels of antibodies or the presence of nonspecific or cross-reacting antibodies or substances. For binding assays, such as an enzyme-linked immunosorbent assay (ELISA), a labeled anti-IgM as the secondary antibody detects only IgM rabies virus antibodies as the primary antibody. The anti-IgM level can be compared to the anti-IgG level in the same sample by performing the same assay, but using a labeled anti-IgG as the secondary antibody. The competitive ELISA method utilizes a competing monoclonal antibody to a rabies protein epitope and measures the ability of antibodies in the test sample (of serum or CSF) to out-compete the monoclonal antibody for attachment to that particular epitope [20]. This makes the assay very specific for measuring antibodies that bind to a specific epitope on a given rabies protein [21]. In addition, variation can be introduced to assay methods to make them easier to perform and easier to standardize for use in very diverse environments and laboratories. For example, molecular techniques involving pseudotyped viruses as vectors, such as the lentiviral vector system, to express the rabies glycoprotein have several advantages. They provide the ability to (a) standardize the target epitope; (b) test for neutralization against multiple strains or genotypes of viruses; (c) use a lower level of biohazard practices (i.e., making them potentially safer than using live rabies virus), and (d) improve the economy of the test methods [22]. These are some of the factors to consider when choosing the method that is “fit-for-purpose.”

Determination of the cut-off value (i.e., the point that indicates seroconversion or determination of adequate vaccination) is specific for each test method and is critical to how results are interpreted. In general, a serum neutralization titer of 1∶100 (90% endpoint titer) is acceptable as effective, even though antibody levels in tissue might actually be lower [23]. The antibody titer (level) of 0.5 IU (international units)/mL, which is recognized globally, was first mentioned in the Eighth Report of the World Health Organization (WHO) Expert Committee on Rabies, 1992. With regard to pre-exposure vaccination, the report states: “All persons who work with live rabies virus in a diagnostic, research or vaccine production laboratory should have a serum sample tested for rabies virus-neutralizing antibodies and a booster administered when the titre falls below 0.5 IU/mL.” The report also recommends that the level of RVNA be determined by the MNT or the RFFIT, using a common challenge virus strain [24]. It is clear that the level of 0.5 IU/mL was established as an indication of adequate vaccination (not protection!) in humans at risk of rabies exposure, and that it refers to using a serum neutralization test with a standard challenge virus strain. In the US, the recommendations of the Advisory Committee on Immunization Practices (ACIP) state “If the titer falls below the minimum acceptable antibody level of complete neutralization at a serum dilution of 1∶5, a single pre-exposure booster dose of vaccine is recommended for persons at continuous or frequent risk for exposure to rabies” (the minimum acceptable antibody level is not defined in IU/mL) and recommend the RFFIT method for testing [25]. Therefore, when other methods are employed to measure rabies-specific antibodies in humans or when the RFFIT is employed for other species (not humans), the accepted level of 0.5 IU/mL may not apply. In a review of rabies challenge studies, valid cut-off values for RFFIT results in cats and dogs of 0.1 and 0.2 IU/mL, respectively, were suggested [2]. The level of 0.5 IU/mL by RFFIT or FAVN methods is recognized by regulatory authorities from most rabies-free areas as proof of adequate response to vaccination for importation of cats and dogs [26]. When results of testing raccoon sera by both RFFIT and a commercial veterinary ELISA kit are compared, similar measures of sensitivity and specificity are obtained only when using different cut-off values (see Table 1). For raccoon sera tested by RFFIT, setting the cut-off level at 0.5 IU/mL allows for the possibility of nonspecific inhibitors to be present in the sera of wildlife, which may give false-positive results if the cut-off level is set too low. Often the ELISA method is less susceptible to interfering substances because the serum dilution used in the assay is generally higher. Measurements above 0.1 EU (equivalent units)/mL appear to be specific for this set of samples, using this particular test kit. When monitoring bait uptake in raccoons in oral baiting programs, it is more important to use a method that estimates levels of potential immunity of a population than to determine the degree of protection in an individual raccoon. Employing different cut-off values in studies when comparing results produced with the same method can result in misleading conclusions. Therefore, the assay and cut-off value that can distinguish vaccinated from unvaccinated raccoons becomes the best “fit-for-purpose” method. Similarly, the cut-off level is significant in a study of the human response to a rabies vaccine that compares three ELISA methods against the RFFIT method utilizing the seroconversion cut-off value of 0.5 IU/mL [27]. Calculations and hence conclusions derived from the seroconversion RFFIT results will vary depending on the cut-off level used, either 0.5 IU/mL or the ACIP-accepted level of complete neutralization at a 1∶5 dilution. For example, determination of sensitivity and specificity measures of the ELISA methods in comparison with RFFIT results, interpreted as negative or positive for seroconversion by the ACIP level, rather than the 0.5 IU/ml level used in the paper, will give different values [27]. In these two examples, the appropriate cut-off value should be determined by and specific to each method, and in relation to the purpose of testing and regulatory requirements. In addition to setting appropriate cut-off levels for particular methods, regulatory agencies, such as the European Pharmacopoeia, the USFDA, or national import authorities in rabies-free areas, may also require laboratory validation and approval or certification.

**Table 1:** Summary of rabies serology results of 100 raccoon subjects (50 vaccinated/50 unvaccinated) comparing RFFIT and ELISA methods.

Cut-off level			Vaccination Status		Specificity	Sensitivity
			Yes	No		
0.1 IU/mL or EU/mL	ELISA Result	# above cut-off	35	0	1.00	0.77
		# below cut-off	15	50		
	RFFIT Result	# above cut-off	42	10	0.83	0.86
		# below cut-off	8	40		
0.5 IU/mL or EU/mL	ELISA Result	# above cut-off	24	0	1.00	0.66
		# below cut-off	26	50		
	RFFIT Result	# above cut-off	32	1	0.98	0.74
		# below cut-off	18	49		

Two cut-off values for seroconversion (0.1 IU/mL and 0.5 IU/mL were used for determination of specificity and sensitivity in relation to vaccination status. Raccoons were wild-caught. Vaccinated raccoons were orally vaccinated with V-RG. Blood samples were drawn at various time-points after vaccination and tested by RFFIT and ELISA (Bio-Rad Platelia Rabies Kit II).

### Method Standardization and Validation

Standardization of rabies serologic test methods is essential to provide a meaningful comparison of antibody characteristics and potency in vaccinated or exposed individuals. This becomes especially relevant when rabies serology results are compared between different laboratories and different studies over time. The fluorescent antibody virus neutralization (FAVN) test was developed in part to address standardization of the various modifications of the RFFIT in use [28]. Variables in the test, which include the challenge virus, antibodies, target cells, and cut-off values, must be standardized for the test results to be comparable. For example, with whole rabies virus versus rabies virus glycoprotein as the antigen in an indirect ELISA test, where all the other variables are standardized, results would not be expected to be comparable. In most test samples, the majority of the rabies virus antibodies being analyzed will be directed against the glycoprotein, but antibodies to nucleoprotein and phosphoprotein antigens of the virus are not uncommon and will be detected in an indirect ELISA based on the whole virus [29]. In the measurement of potency of monoclonal antibodies, it is important to consider that the specificity of an antibody for a single epitope may not be sufficient to neutralize all epitope (antigenic) variations of a challenge virus strain or even a number of quasispecies within a single virus stock [30],[31]. Similarly, different challenge virus strains can influence the results obtained in serum neutralization testing. In a study of human subjects that compared serum titers measured in vitro against challenge virus strains that were either homologous or heterologous to the vaccine strain revealed that, in the majority of subjects, higher titers were detected against the homologous strain [32]. Also, both high and low challenge virus doses can affect the determination of rabies Ig potency [33]. The use of a recognized standard anti-rabies serum control as a reference will standardize the results of a method and allow for conversion of the measurements (e.g., titers, optical density readings, etc.) into recognized units such as IU/mL or EU/mL.

The international reference standards for RIG products in use today include of the WHO first International Standard for Rabies Immunoglobulin, the WHO second International Standard for Rabies Immunoglobulin, and the Office International des Épizooties (OIE) canine RIG reference serum. The potency of each of these products has been assigned a value by serum neutralization methods [34]–[36]. The original international standard RIG reference serum, which was of equine origin, was established in 1955. Based on the lyophilized product weight of 86.6 mg per ampoule, a potency value of 86.6 IU was assigned. The WHO first RIG reference serum of human origin was prepared from pooled sera from vaccinated humans. The human RIG potency was established by interlaboratory testing against the standard equine RIG in a RFFIT performed in six laboratories. The test involved two dilutions (low and high) of the product in four replicate assays. After statistical analysis, a potency of 59 IU was assigned in 1984 to the WHO first International Standard for RIG. In a similar manner, a second pool of sera from vaccinated humans was tested in 1993, assigned a potency of 30 IU, and established as the WHO second International Standard for RIG reference serum. The RIG reference serum used in the US, Lot R-3, is a portion of the lot that became the WHO first International Standard for RIG reference serum. The OIE canine RIG reference serum has a potency of 6.7 IU, and batches of this product are calibrated against the WHO second International Standard for RIG reference serum. The relative potencies of these two WHO human RIG reference sera were compared by the USFDA in 1997 and again by Kansas State University Rabies Laboratory in 2006. This comparison showed a decrease in the relative potency of WHO first International Standard in relation to the potency of WHO second International Standard of 2.5% in 1997 and 14% in 2006. These differences are not considered statistically significant, but they illustrate the effect of using different reference standards when comparing rabies serology results and the importance of quality control monitoring and standards.

**Figure 1:**
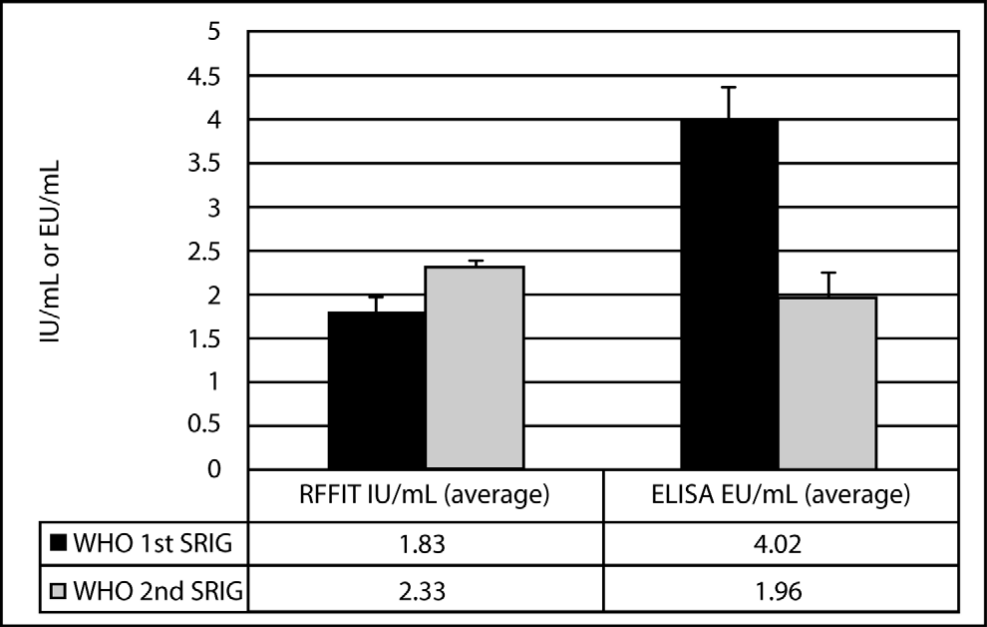
International standard RIG reference sera. WHO first SRIG and WHO second SRIG were diluted to 2.0 IU/mL according to the potency as labeled. The SRIG preparations were evaluated in three independent test batches by both RFFIT and ELISA (Bio-Rad Platelia Rabies Kit II) methods. The IU/mL value was calculated against the WHO first SRIG and the EU/mL was calculated against the kit standard. Displayed are the average IU/mL or EU/mL values with one standard deviation.

The use of potency values for RIG that are assigned by serum neutralization methods as opposed to those assigned by binding assays is inappropriate and problematic. Figure 1 illustrates the discrepancies that can occur when various RIG reference sera of the same potency determined by serum neutralization are used to calculate potency values obtained by an ELISA method. When the WHO first and second RIG reference sera are diluted to 2.0 IU/mL to give similar potency values by RFFIT, the ELISA results are discrepant. Thus, if the WHO first RIG is used as the reference control for the standard curve in an ELISA assay, lower results will be obtained than if the WHO second RIG is used.

“Method-of-result” calculation is an additional factor to consider in the standardization of an assay. For example, the use of either 50% or 100% endpoint titers, both of which can be calculated for serum neutralization assays, will yield different titer values. For any comparison of results, it is essential to know, and should always be stated, what calculation was used to generate titer values.

## Conclusions

It is important that characteristics and variability associated with different assay methods that define antibody titers continue to be defined as we advance our understanding of immunity and disease prevention. The measurement of rabies specific antibodies in vitro is essential and should be the first step taken to establish whether rabies immunity following vaccination is successful. Even so, in vitro measurements may never completely correlate with what may be regarded as protective in vivo. Even use of “gold standard” measurements of rabies virus neutralizing activity in vitro provides only an estimate of protection. Despite the long history of using virus neutralization tests, there are still no internationally recognized standard protocols for measuring rabies-specific antibodies, for specifically defining RIG potency for human rabies biologics formulation, or for assessing oral rabies vaccine uptake in wildlife. Method standardization requires the careful examination and evaluation of laboratory test performance, which may include audits of laboratory operations, the publication and sharing of standard operating procedures, and inter- and intralaboratory evaluation of proficiency in performing tests. Proficiency testing, training, and certification, as well as trends in assay performance are components of quality assurance programs to ensure continuing adherence to acknowledged and accepted standards. These efforts will require the collaboration of all organizations performing tests, including national laboratories, regulatory agencies, commercial companies, and rabies diagnostic and research laboratories.

For a fatal disease such as rabies, where vaccination and passive immunity (use of HRIG) are absolutely required for protection upon exposure, verification of the accuracy and efficacy of every assay involved in predicting the relevant in vivo protection against the virus is vital. Moreover, there is a basic requirement to confirm the appropriateness and applicability of any neutralization test system or antigen binding assay used, as a predictor of in vivo protection, with each new type of prophylactic biologic, be it a vaccine, Ig, or monoclonal antibody formulation. In the case of vaccines, it is imperative to understand how antibodies neutralize viral infectivity in order to have input into the design and presentation of immunogens of the vaccine that elicit the specificity and isotype of antibody produced to confer the maximum neutralizing, and ultimately, protective activity.

**Box 1. Learning Points**

1. RVNA have been demonstrated to be critical for protection against rabies. Even so, in vitro measurements are only a partial determination of the degree of protection provided in vivo. Not all methods that measure rabies-specific antibodies will determine the neutralizing function of the antibodies.

2. In the selection of the most appropriate assay for rabies antibody detection, consideration of the purpose and use of the results is as important as the established performance characteristic of the assay.

3. Standardization of assays includes both assay components and test conditions. Alterations will cause variation in results; therefore use of a particular assay (e.g., ELISA or serum neutralization) does not guarantee comparable results if the assays have not been standardized.

4. Because rabies is a fatal disease for which development of a sufficient RVNA response is paramount for protection, assay specificity, sensitivity, and accuracy must be verified for meaningful clinical decisions to be made based on the results.

5. Steps toward better understanding and use of rabies serology assays will include collaboration of national laboratories, regulatory agencies, as well as commercial and research laboratories. Greater cooperation and standardization of rabies serology assays will lead to increased understanding of the relationship between in vitro measurement and in vivo protection.

**Box 2. Key References in the Field**

1. Smith JS, Yager PA, Baer GM (1973) A rapid reproducible test for determining rabies neutralizing antibody. Bull World Health Organ 48: 535–541.

2. Cliquet F, Aubert M, Sagne L (1998) Development of a fluorescent antibody virus neutralisation test (FAVN test) for the quantitation of rabies-neutralising antibody. J Immunol Methods 212: 79–87.

3. Aubert MF (1992) Practical significance of rabies antibodies in cats and dogs. Rev Sci Tech 11: 735–760.

4. Grassi M, Wandeler AI, Peterhans E (1989) Enzyme-linked immunosorbent assay for determination of antibodies to the envelope glycoprotein of rabies virus. J Clin Microbiol 27: 899–902.

5. Irie T, Kawai A (2002) Studies on the different conditions for rabies virus neutralization by monoclonal antibodies #1-46-12 and #7-1-9. J Gen Virol 83: 3045–3053.

## Supporting Information

Alternative Language Abstract S1 French translation of the abstract by Celine Jiron Corrales.
(0.03 MB DOC)
