# Supplementary material for: Rabies-Specific Antibodies: Measuring Surrogates of Protection against a Fatal Disease
Source: PLoS Negl Trop Dis. 2010 Mar 9;4(3):e595. doi: 10.1371/journal.pntd.0000595 (PMC2834733; doi:10.1371/journal.pntd.0000595)
Supplement: Alternative Language Abstract S1 — French translation of the abstract by Celine Jiron Corrales. (0.03 MB DOC) [file pntd.0000595.s001.doc]

Rabies Specific Antibodies: Measuring Surrogates of Protection Against a

Fatal Disease

Susan M. Moore and Cathleen A. Hanlon

**Abstract translation from English into French by Celine Jiron Corrales, Research Assistant, Rabies Laboratory, KSVDL, Manhattan, Kansas 66502**

Les anticorps jouent un rôle principal dans la prophylaxie contre de nombreux agents infectieux. Tandis que la neutralisation est une fonction principale des anticorps, les activités de Fc- et du complément-dépendent des protéines multifonctionnelles peuvent aussi être important dans leur protection contre la plupart des virus. La protection contre les pathogènes viraux *in vivo* est complexe, et tandis que la neutralisation du virus – la capacité de l’anticorps d’inactiver l’indice d’infection du virus, souvent mesuré *in vitro* – est importante, elle souvent contribue seulement à une protection partielle. La technique Rapid Fluorescent Focus Inhibition Test (RFFIT) est toujours la meilleure des techniques employées pour mesurer la neutralisation des anticorps antirabique. En plus de mesurer la neutralisation, l’activité de l’antigène spécifique antirabique de l’anticorps peut être aussi mesurée par des techniques immuno-absorbant (les ELISA), en plus d’autres méthodes. Comme pour toutes les maladies, le choix de la technique idéale pour évaluer le titre en anticorps protecteurs, la validation des tests et leurs interprétations sont des facteurs importants à considérer, mais pour une maladie fatale comme celle de la rage, ces facteurs sont d’une importance capitale. Les limitations innées d’un test de laboratoire unidimensionnel pour la mesure de l’anticorps antirabique, en plus de la validation de la méthode de choix, doivent être considérées soigneusement dans la sélection de la méthode du test et dans l’évaluation des résultats qui peuvent être interprétés comme une protection subrogée.
